# Effects of hypodontia on craniofacial structures and mandibular growth pattern

**DOI:** 10.1186/1746-160X-7-23

**Published:** 2011-12-06

**Authors:** Amelia Kreczi, Peter Proff, Claudia Reicheneder, Andreas Faltermeier

**Affiliations:** 1 Department of Orthodontics, University Clinics, Franz-Josef-Strauss-Allee 11, D-93042 Regensburg, Germany

**Keywords:** hypodontia, mandibular growth, missing teeth

## Abstract

**Introduction:**

This study was performed to examine craniofacial structures in persons with hypodontia and to reveal any differences, that may occur, when agenetic teeth are only found in the maxilla, the mandible or in both jaws. The groups consistent of 50 children (33 girls, 17 boys) aged between 9 and 13.5 years were analyzed and assigned to three subgroups. Group 1 = upper jaw hypodontia. Group 2 = lower jaw hypodontia. Group 3 = hypodontia in both jaws.

**Materials and methods:**

Eleven angular and three index measurements from lateral encephalographs and two linear measurements from dental blaster casts were calculated. All data was statistically analyzed, parameters with p < 5% were investigated for each subgroup respectively.

**Results:**

In comparison with standards the study group showed bimaxillary retrognathism and a reduction of the lower anterior facial height. Moreover both overbite and overjet significantly increased. Other values laid within the normal ranges. Evaluating results of the subgroups, differences in the means of SNA, SNB and overjet between the groups were observed.

Analysis of the mandibular growth pattern revealed, that neither vertical nor horizontal patterns are dominant in hypodontia patients.

**Conclusions:**

In certain dentofacial parameters differences between persons with hypodontia and such with full dentition exist. According to our findings agenetic teeth may have a negative influence on the saggital development of a jaw and the lower face and may be responsible for increased overbites. This should receive attention in orthodontic treatment of hypodontia patients.

## Introduction

Congenital missing teeth are a common anomaly in the craniofacial skeleton. The prevalence of dental agenesis varies dependent on continent, race and gender as a meta analysis by Polder et al. [1] reveals. In white Europeans a total prevalence of 5.5 percent was found in permanent dentition, not including the third molar. The number of missing teeth in the maxilla was comparable with that in the mandible. Several studies confirm, that females are concerned more frequently from this anomaly than males [1-3]. Excluding the third molar the most common teeth showing agenesis are the mandibular second premolar and the lateral maxillary incisor [4,5]. Whereas in maxillary lateral incisors bilateral agenesis occurs more often, unilateral agenesis is more common in other teeth [1]. Dependent on the number of agenetic teeth, hypodontia, oligodontia and anodontia can be differentiated. The maijority of persons with hypodontia suffers from only one or two missing teeth [1,6].

Oligodontia is described as very heterogeneous [7] and rather rare (0.6-0.7 percent) [8]. Moreover taurodontism, reduced tooth length and delayed tooth formation were observed in relationship with this anomaly [9]. It has been emphasized, that especially persons with more severe hypodontia should be closely surveyed for syndromal illnesses such as ectodermal dysplasia, because with the number of agenetic teeth also the probability, that hypodontia is part of a sydrome increases [10].

However hypodontia also exists as isolated condition. In their recent study De Coster et al. reported [11], that hypodontia shows a genetically and phenotypically heterogeneity and most frequently results from various gen mutations. Further it was observed, that the incidence of agenetic permanent teeth has increased in the Caucasian population over the last century [3]. Hobkirk and Brook [10] surveyed their patients in a multidisciplinary clinic for the management of hypodontia in Newcastle and revealed, that the most common complaints were poor appearance and lack of function. Apart from that, alternations in the craniofacial morphology may be relevant for orthodontic treatment of hypodontia patients. Possible reasons for a relationship between hypodontia and skeletal structures are, among others, the fact that teeth serve as functional units, whereby local bone growth is stimulated [12]. It can therefore be hypothesized, that congenital missing teeth cause underdevelopement of the jaw basis. This theory is stregthend by findings of bimaxillary retrognathism [13], reduced maxillary and mandibular length [14] and more backward chins [15]. In contrast to this, several studies reveal more prognathic mandibles [16,17]. It was suggested, that severe hypodontia causes a lack of occlusal support, which results in an underdevelopment of the lower face and anterior rotation of the mandible, leading to prognathism of the lower jaw [16]. Øgaard and Krogstad [15] confirmed this, finding a decrease of mandibular plane angle and a reduced anterior facial height in persons with more than 10 congenital missing teeth. The reduction of the anterior facial height is a common report in studies on hypodontia, but wheter it results from a reduction in the upper facial height [5], the lower facial height [18] or both [14] is dicussed controversially. Despite these relevant observations, both Yüksel and Ücem [19], who examined the effects of hypodontia dependent on the location of the missing teeth and Øgaard and Krogstad [15] come to the conclusion, that tooth agenesis has little effect on the cranifacial growth pattern. In accordance with this, the recent study of Bauer et al. [18], who investigated the general growth pattern according to Segner [20] and Hasund [21], failed to reveal statistically relevant correlation between craniofacial growth pattern and the congenital absence of certain permanent teeth. Alternatively to an unique growth pattern, typical dentofacial structures in persons with hypodontia may be due to a dental and functional compensation [15]. Especially various malpositions of incisors were attributed to functional alternations, such as imbalance of lip-tongue pressure [19].

Little consent about the influence of hypodontia on the facial skeleton is found in literature. More research is required on this subject and hence our aim was to investigate craniofacial morphology of individuals with non-syndromic hypodontia in a german population. While it has been examined, whether the tooth type (anterior and posterior hypodontia) and the number of agenetic teeth (mild, moderate and severe hypodontia) play a role in considering morphological characteristics, none of the recent studies seems to investigate the effects of hypodontia for each jaw respectively. Therefore we specified significant results obtained from a sample with randomly distributed agenetic teeth in forming three subgroups and investigate the effects of upper jaw hypodontia, lower jaw hypodontia and both jaw hypodontia respectively. Modified standard values for Regensburg following norms published by Segner [20] and Hasund [21] severd as controls. The general mandibular growth pattern analyzed according to Björk [22] was also objective of this study.

## Materials and methods

**Figure 1:**
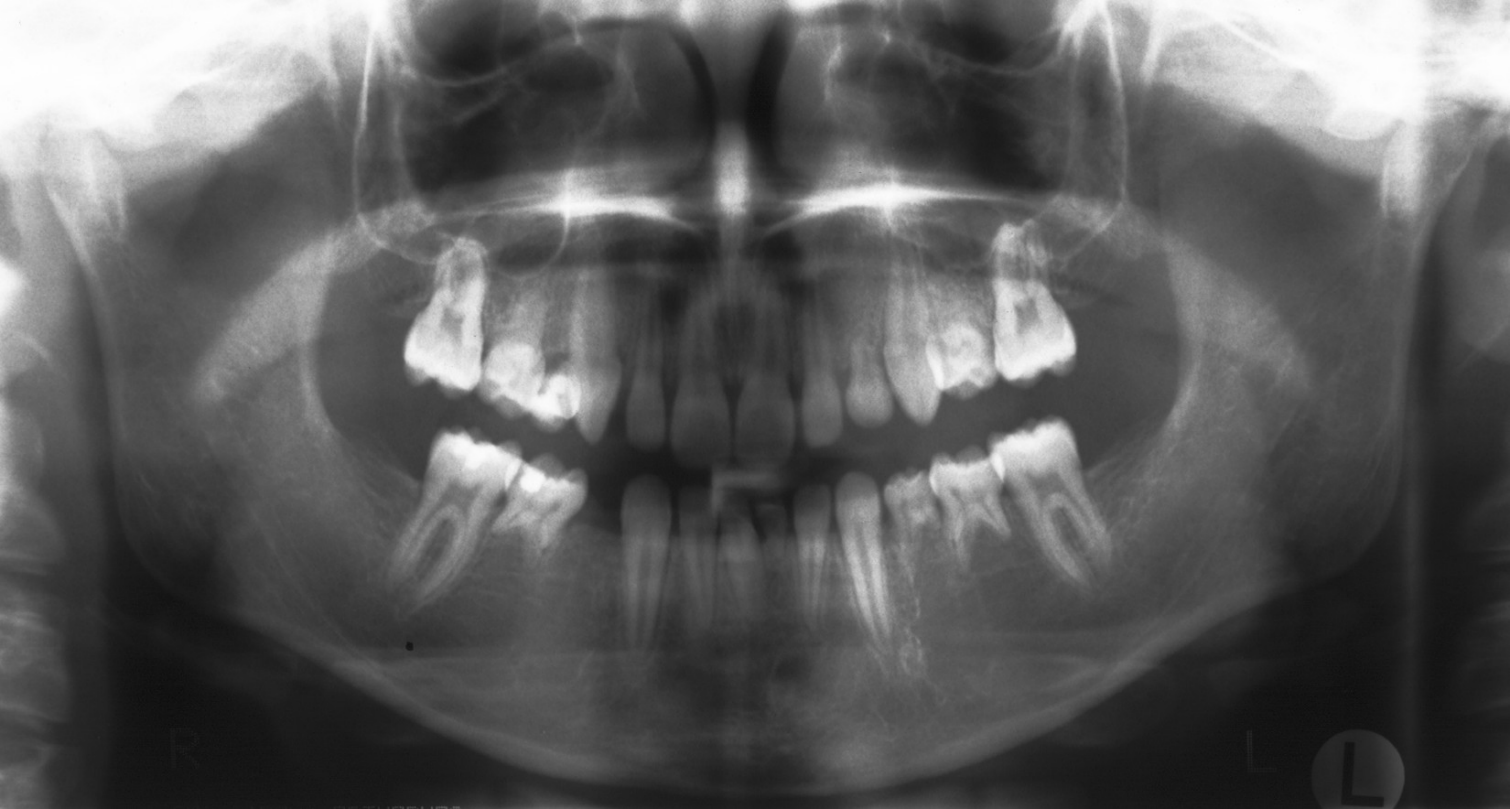
**Radiographs of a person with 13 congenital missing teeth: Orthopantomogram**.

**Figure 2:**
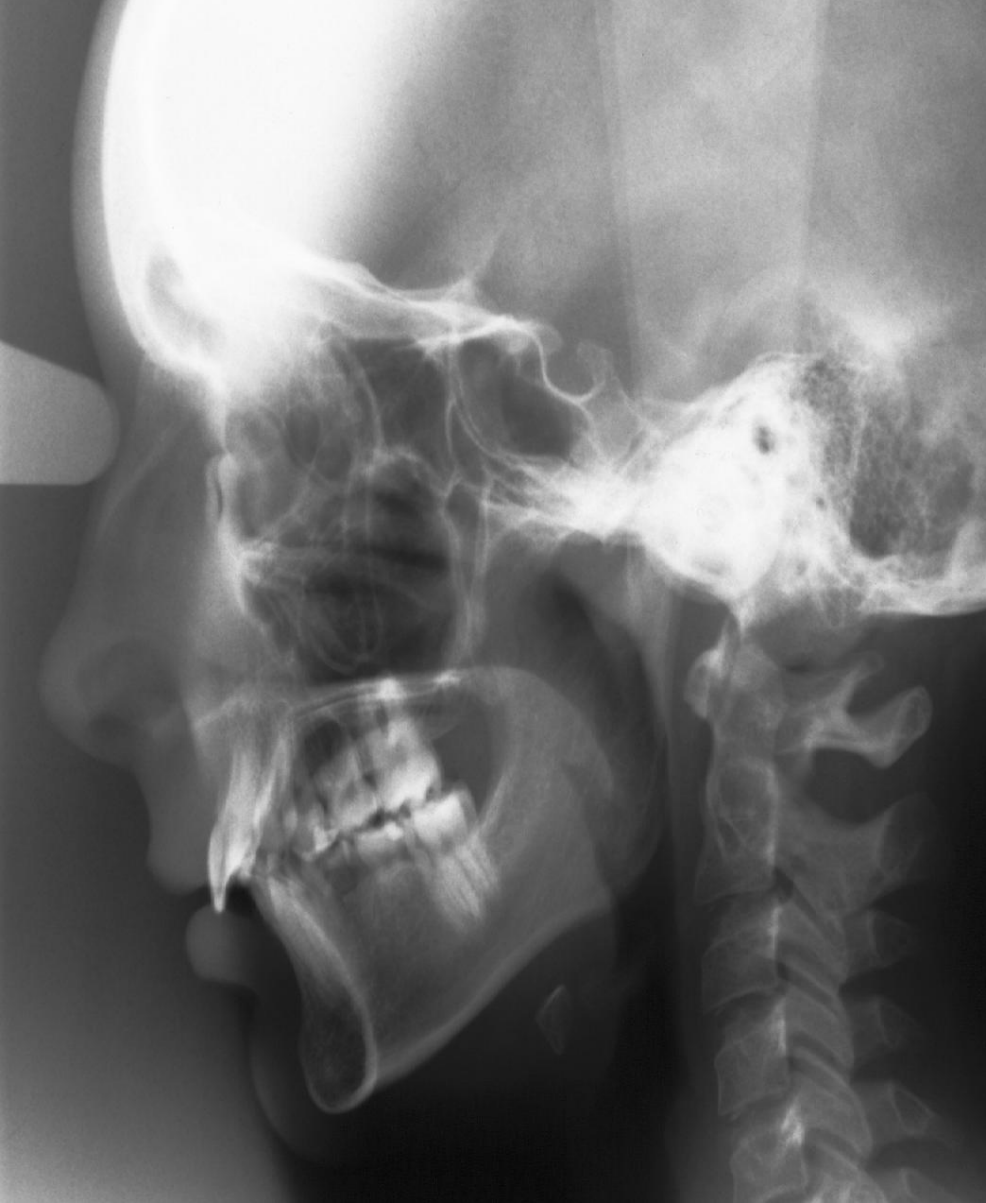
**Radiographs of a person with 13 congenital missing teeth: lateral encephalometric radiograph**.

The material for this retrospective statistic comprised orthopantomograms, lateral cephalometric radiographs and dental plaster casts of 50 children with at least two congenitally missing teeth in one jaw. The data was collected from 17 boys and 33 girls aged between 9 and 13.5 years (mean 11,5 years) and prior to any orthodontic treatment. Children with ectodermal dysplasia, cleft lip and palate, or other craniofacial anomalies were not included in the study group. Figure 1 and 2 show an orthopantomogram (1) and a lateral encephalometric radiograph (2) of a person with 13 congenital missing teeth.

**Figure 3:**
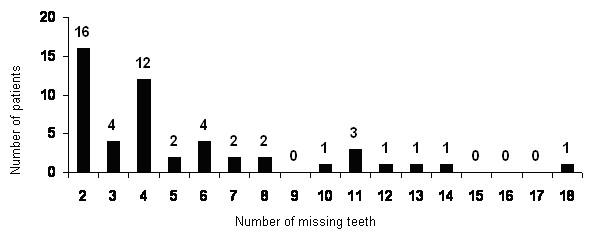
**Survey of number of agenetic teeth in the sample (N = 50)**.

The number of missing teeth in each subject was recorded from orthopantomograms and verified by anamnesis and clinical examination, both documented in each patient's file. It ranged from 2 to 18 teeth with a mean value of 5 missing teeth per person (Figure 3). The lateral cephalometric radiographs were taken in a multigraph (Siemens, Germany, focus-film-distance 4.0 m).

**Figure 4:**
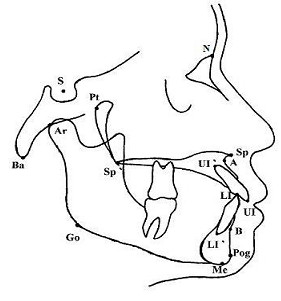
**Landmarks for analysis of lateral encephalographs**. S, sella turcica; N, nasion; Ba, basion; Go, gonion; Me, menton; Pog, pogonion; B, B-Point; A, A-Point; LI', lower incisor rout edge; LI, lower incisor crown edge; UI, upper incisor crown edge; UI', upper incisor rout edge; Sp, anterior nasal spine; SP', posterior nasal spine; Pt, pterygomaxillary fissure; Ar, articulare.

All reference points were manually scanned and digitized by a single investigator using a numonics lightbox. Landmarks are shown in Figure 4. Eleven angular and five linear measurements were calculated automatically by the computer program Ratisbona (Dentofacial planer Version 7.02).

Overjet (saggital) and Overbite (vertikal) were measured with a caliper in blaster casts, manufactured at the same time as the x-rays were taken.

Angular mesurements in degrees (°):

saggital: ∠SNA: inclination of maxilla to skullbase

∠SNB: inclination of mandibule to skullbase

∠ANB difference: saggital jaw relationship (0.4 × SNA+0.2 × ML-NSL- 35.16 = individual ANB; indiv.ANB-ANB = ANB difference)

vertikal: ∠NL-ML: vertical jaw relationship

∠NL-NSL: maxillary plain inclination

∠ML-NSL: mandibular plane inclination

∠Gn-Pt/Ba-N: facial axes

∠ArGoMe: gonionangle

dental: ∠UI/NA: inclination of upper incisor

∠LI/NB: inclination of lower incisor

∠UI/LI: interincisal angle

Linear measurements in mm:

Saggital: Wits value: saggital distance A-B projected on the occlusal plane

Dental: Overjet (saggital)

Overbite (vertical)

Indexes: Hasund index: upper to lower anterior facial height (N-SP'x100/SP'-Me)

Jarabak index: posterior to anterior facial height (S-tgo × 100/N-Me)

Statistical methods:

All statistical analysis were done using SPSS (Statistical Package for Social Sciences, Chicago, IL, USA) version 15.0 for windows.

The results were calculated with the student's t-test for paired samples. In case the p-value was < 0.05 the difference between our distribution and the distribution of the equivalent standart value was considered to be statistically significant.

After analysing the parameters stated abouve for the total examination group, persons were asigned to three subgroups:

Group 1: Two or more congenitally missing teeth in the maxilla (11 subjects).

Group 2: Two or more congenitally missing teeth in the mandible (12 subjects).

Group 3: Two or morge congenitally missing teeth in both jaws (27 subjects).

In case a singel tooth was missing in one of the jaws, it was not taken into consideration in this management.

The data of each group was analyzed seperately with the statistical methods stated abouve. However only parameters that showed a significance level of at least 5% in the first analysis were considered (SNA, SNB, Hasund index, overjet, overbite).

**Figure 5:**
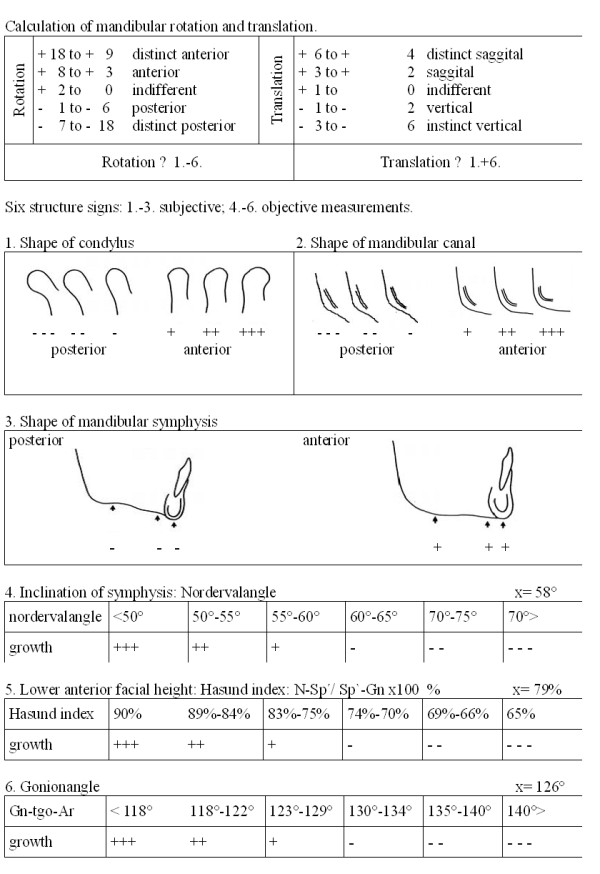
**Mandibular growth pattern analyses according to Björk (1968)**.

To investigate on the general mandibular growth pattern, lateral encephalographs were examined by a single investigator according to Björk [22]. This method is established on the basis of six mandibular structure signs, three of them objective measurments, the others subjective parametres. The gonionangle, the nordervalangle and the hasund index were calculated for each person by methods explained earlier in the text. The shape of the condylus, the mandibular canal and the mandibular symphysis were assessed using a lightbox and a table with reference shapes as shown in Figure 4. Each parameter was appraised with a score ranging from three minus to three plus. Minus indicating vertical growth and plus indicating horizontal growth. The mandibular growth patterns is characterized by two components: the translation and the rotation. In accordance with Björk, the shape of the condylus and the gonionangle determined the translation, and the scores for all six parameters together determined the rotation of the mandible (Figure 5).

## Results

In our study the most frequent tooth missing was the lower second premolar (27%), followed by the upper lateral incsisor (17%) and the upper second premolar (15%). In the upper jaw hypodontia group (1) 38% incisor agenesis and in the lower jaw hypodontia group (2) 80,7% premolar agenesis was found. Table 1 shows prevalences of dental agenesis for all tooth types.

**Table 1:** Distribution of agenetic teeth according to thooth type in the study group (50 people).

Upper jaw	Tooth type	17	16	15	14	13	12	11	21	22	23	24	25	26	27
	Number of missing tooth	4	2	20	4	3	22	1	1	22	5	6	19	2	6
Lower jaw	Tooth type	47	46	45	44	43	42	41	31	32	33	34	35	36	37
	Number of missing tooth	8	1	35	5	3	6	12	12	6	3	6	35	1	6

Our sample comprised 33 females and 17 males. Hence approximately twice as many females were effected than males. In angular and linear measurements significant associations between mean values of the examination group compared to standart values were observed. In the saggital plain both the maxilla and the mandible showed a retrognathic inclination to the skullbase (reduced ∠SNA and ∠SNB). Further the Hasund index between upper and lower anterior facial height increased. Analysis of the dental parametres revealed significantly increased vertical overbite and saggital overjet (Table 2).

**Table 2:** Comparison of means (± standart deviations) in the study group (N = 50) and the control standart means (± standart deviation) including the respective p-values of the t-test.

Values	Mean values ± SD	Norms ± SD	Mean difference	p-value
**∠SNA (°)**	80.28 ± 3.78	82.00 ± 3.00	-1.72	0.002**
**∠SNB (°)**	77.32 ± 4.31	80.00 ± 3.00	-2.68	0.000**
**∠indiv.ANB (°)**	-0.76 ± 3.10	0.00 ± 2.00	-0.76	0.091
**Wits appraisal (mm)**	-6.0E-03 ± 3.69	0.00 ± 1.00	-6.0E-03	0.991
**∠ArGoMe (°)**	126.89 ± 8.50	126.00 ± 6.00	0.89	0.464
**∠Gn-Pt/Ba-N (°)**	90.17 ± 5.61	90.00 ± 3.00	0.17	0.829
**Hasund index**	86.77 ± 8.66	79.00 ± 5.00	7.77	0.000**
**Jarabak index**	62.88 ± 5.59	63.50 ± 1.50	-0.62	0.438
**∠ML-NL (°)**	25.36 ± 6.85	23.50 ± 6.00	1.86	0.061
**∠ML-NSL (°)**	33.62 ± 6.93	32.00 ± 6.00	1.62	0.105
**∠NL-NSL (°)**	8.48 ± 4.16	8.50 ± 3.00	-2.00E-02	0.973
**∠UI/ML (°)**	72.08 ± 10.40	70.00 ± 5.00	2.08	0.168
**∠LI/NL (°)**	92.68 ± 8.56	92.00 ± 6.00	0.68	0.592
**∠UI/LI (°)**	132.99 ± 11.70	132 ± 6.00	0.99	0.569
**Overbite (mm)**	3.86 ± 1.51	2.00 ± 1.00	1.86	0.000**
**Overjet (mm)**	2.73 ± 1.93	2.00 ± 1.00	0.73	0.021*

(In case of p < 0.05 the difference in values becomes significant).

(∠SNA, ∠SNB, Hasund ratio, overbite and overjet).

* p = significant at the 5% level.

** p = significant at the 1% level.

The statistical analysis showed no significant difference in the values: individual ANB, Wits appraisal, ∠ArGoMe, ∠Gn-Pt/Ba-N, Jarabak index, ∠ML-NSL, ∠NL-NSL, ∠ ML-NL, ∠UI/NA, ∠LI/NB and ∠UI/LI. All results are shown in Table 2.

In the evaluation of the subgroups only parameters, that revealed significant associations in the first analyses were taken into consideration. The results show, that in each group the Hasund index and the overbite significantly increased.

In Group 3 bimaxillary retrognathism could be revealed, while in group 2 only the mandible showed a retrognathic inclination. Group 1 had neutrally inclined mandibles and retrognathic maxillas, altough the difference in values was not statistically significant. An increased overjet was only found in group 2. Results are listed in Table 3. Analysis on the general mandibular growth pattern according to Björk's method, mostly revealed indifferent patterns in the hypodontia sample. An approximately even distribution between vertical and horzontal patterns was found for both the rotation and translation component shown in Table 4 and 5.

**Table 3:** Analysis of significant results from table 1 for each subgroup seperately.

	Variable	Mean values ± SD	Norms ± SD	Mean difference	p-Value
Group 1: Upper jaw hypodontia	**SNA (°)**	79.93 ± 3.90	82.00 ± 2.00	-2.07	0.109
	**SNB (°)**	78.00 ± 5.70	80.00 ± 2.00	-2.00	0.272
	**Hasund index**	88.24 ± 9.19	79.00 ± 5.00	9.24	0.008**
	**Overjet (mm)**	1.87 ± 1.95	2.00 ± 1.00	-0.13	0.842
	**Overbite (mm)**	3.94 ± 1.61	2.00 ± 1.00	1.94	0.003**
Group 2 Lower jaw hypodontia	**SNA (°)**	80.16 ± 4.32	82.00 ± 2.00	-1,84	0.168
	**SNB (°)**	76.49 ± 4.12	80.00 ± 2.00	-3.50	0.013*
	**Hasund index**	85.63 ± 5.87	79.00 ± 5.00	6.63	0.002**
	**Overjet (mm)**	3.00 ± 0.93	2.00 ± 1.00	1.00	0.003**
	**Overbite (mm)**	3.60 ± 1.10	2.00 ± 1.00	1.60	0.001**
Group 3: both jaw hypodontia	**SNA (°)**	80.47 ± 3.62	82.00 ± 2.00	-1.53	0.037*
	**SNB(°)**	77.42 ± 3.84	80.00 ± 2.00	-2.58	0.002**
	**Hasund index**	86.68 ± 9.62	79.00 ± 5.00	7.68	0.000**
	**Overjet (mm)**	2.88 ± 2.21	2.00 ± 1.00	0.88	0.083
	**Overbite (mm)**	3.90 ± 1.64	2.00 ± 1.00	1.90	0.000**

Mean values (with standart deviation), mean difference to control standart means and respective p-values (∠SNA, ∠SNB, Hasund index, overbite and overjet).

* p = significant at the 5% level.

** p = significant at the 1% level.

**Table 4:** Rotation of the mandible, calculated by the shape of the condylus, the mandibular canal and symphysis, the nordervalangle, the hasund index and the gonion angle according to Björk.

posterior															indifferent																anterior						
8																																					
7																																					
6																	•										•										
5																																					
4																										•											
3													•		•			•		•	•		•														
2																•			•					•	•				•								
1	•								•	•				•														•		•							

x-axes: direction of mandibular rotation. y-axis: number of persons (∑ = 50).

**Table 5:** Translation of mandible in persons with hypodontia calculated by the shape of the condylus and the gonion angel according to Björk.

vertical						indifferent					anterior		
12													
11							•						
10													
9									•				
8					•						•		
7													
6													
5													
4													
3						•				•			
2		•	•	•									
1								•				•	

x-axis: direction of translation. y-axis: number of persons (∑ N = 50).

## Discussion

At the University of Regensburg computer based analysis of lateral encephalographs are performed with the help of the program Ratisbona (Dentofacial Planner Version 7.02). For evaluation norms published by Segner [20] and Hasund [21] are used. These standard means seemed most suitable to serve as controls in our study, as they represent a large local population. However one disadvantage of our method is, that all persons regardless of age or gender were considered with the same standard values. This is partly compensated by the rather homogeneous age distribution, ranging from 9 to 13.5 years. In a longitudinal study of Roald and Wisth [23] 9 year old children showed the same morphological differences at the age of 16 compared to controls with complete dentition. Moreover, at this age no gender dimorphism could be revealed in cranifacial characteristics relevant for our investigations [15,16]. Therefore it seemed justified to pool the material of both sexes to enlarge the sample size. Nevertheless, linear measurements from lateral encephalographs were avoided, as differences in gender and age distribution could distort the results. Persons with prior orthodontic treatment, such with cleft lip and palate or syndromal illnesses were not included in the sample. These measure was taken to avoid circumstances, that other than hypodontia itself, may influence the craniofacial morphology and bias results that focuse on the effects of tooth agenesis. To increase the severety of hypodontia in the total sample and to achieve greater differences between the supgroups we only included persons with a minimum of two congenital missing teeth in one jaw.

Our sample comprised approximately twice as many females than males and so confirms reports on a higher prevalence for tooth agenesis in females [1]. A ratio of 2:1 was found earlier in a german population by Bauer et al. 2009 [18].

In accordance with the meta-analysis of Polder et al. [1] it could be shown, that the lower second premolar, followed by the upper lateral incisors and the upper second premolars were most frequently missing, whereas the lower first molars and the upper central incisors were the least effected tooth types.

Results obtained from the statistical analyses, showed several significant associations between norms and our hypodontia samples: The inclination of the maxilla in the saggital plain was significantly retrognathic regarding to the skullbase compared to persons without missing teeth. This was also found by Roald et al. [23] and Sarnäs and Rune [24]. In the analysis of our subgroups we investigated a reduced SNA angle in group 1 (upper jaw hypodontia) and group 3 (both jaw hypodontia). Altough the upper jaw hypodontia group showed the smallest SNA means, we failed to achieve statistical significance, as the small sample size could not compensate for the range of this value. Our results agree with the findings of Wisth et al. [5], who reported a significantly reduced SNA angle in persons with upper jaw hypodontia, whereas Øgaard and Krogstad [15] only found the same characteristic in persons missing at least ten teeth. Based on these results it seems likely, that agenetic teeth in the maxilla are responsible for a reduction in maxillary prognathism.

In our study also the mandible reveals a retrognathic inclination to the skullbase (reduced SNB angle). Lisson and Scholtes [14] stated the opposite, while others authors [19] found no significant reduction of the SNB angle. This conflict is most likely due to the great variations in the SNB angle in controls: 79.05° [17]; 75.39° [22]; 80.0° norms by Segner [20] and Hasund [21]. Evaluation of the SNB in the subgroups show, that only persons with missing teeth in the mandible (group 2 and 3) have significant smaller SNB angles. Reduced prognathism of a jaw occurs mainly in that jaw, which is concerned from tooth agenesis. It was suggested before, that a lack of bone apposition associated with the eruption of teeth is responsible for a reduced maxillary length [25]. Based on our results, it seems possible, that agenetic teeth and thus the absence of functional units in a jaw, are jointly responsible for saggital underdevelopment of eighter jaw, demostrated by retrognathism.

Considering the saggital jaw relationship, the individual ANB angle as well as the Wits value laid within the normal range, both indicating a skeletal Class 1 relationship. This agrees with the findings of Dermaut et al. [4] and Yüksel and Ücem [19], who also found Class 1 skeletal relationships most frequently in persons with hypodontia. Following the theory of retrognathism in a jaw with agenetic teeth, the saggital jaw relationship should increase for persons with lower jaw hypodontia and decrease for persons with upper jaw hypodontia. However the correspondent values were not significant in the random sample and therefore not further investigated on in this study.

A decrease in vertical jaw relation and mandibular plane inclination, as it was observed by Nodal et al. [16] and Øgaard and Krogstad [15] in persons with severe hypodontia, could not be found in our study group. It is assumed, that an anterior rotation of the mandible is attributed to a lack in occlusal support, arising from a severe number of agenetic teeth. The majority of persons in our group showed less than 5 missing teeth, which is unlikely to cause a lack of occlusal support and hence an anterior rotation of the mandible. Therefore, we suppose this conflict originates in basic differences in the composition of samples.

Despite the fact that no anterior rotation was observed, the lower anterior facial height significantly decreased in relation to the upper anterior facial height. The same observation was reported by Bauer et al. [18]. The Hasund index increased in the total examination group as well as in each of the three subgroups, obviously regardless of whether hypodontia was present in the mandible the maxilla or both jaws. Based on linear measurements, without calculating any ratio, Lisson and Scholtes [14] reported reduced upper and lower anterior facial heights, whereas Woodworth et al. [17] only found a reduction in the upper anterior facial height. However our findings coincide with most authors observations of a reduced lower facial height only [15,18]. The ratio of posterior to anterior facial height (Jarabak index) did not differ from that of standard controls, thus implying a reduction of the posterior facial height to a similar extent to that of the anterior facial height.

Results obtained from dental measurements in blaster casts showed an increased overbite, as well as a slightly increased overjet. In the whole examination group, as well as in each subgroup, the average overbite nearly doubled compared to standard controls. This is a common finding as similar values (3.7 mm) were published by Chung et al. [26] and further also Dermaut et al. [4] observed deep bite cases more frequently in persons with tooth agenesis compared to controls. Less conspicious was the incease of the overjet. Persons with upper jaw hypodontia showed normal overjets, while persons with lower jaw hypodontia showed the most signifficant increase of this value. This could be attributed to the more retrognathic mandibles in group 2.

In our study little difference in the angulation of incisors or the interincisal angle, compared to controls, could be evaluated. The upper incisors were slightely retroclined, while the lower incisors were neutrally inclined, which resulted in a little increase of the interincisal angle. The values for the relevant parametres were within wide ranges and showed large standard deviation. Two studies [15,25] emphasized a retroclination of upper and lower incisors and consequently an increased interincisal angle. Conversely, a significant protrusion of upper incisors together with a decreased interincisal angle was published by other authors [5,19]. Although malpositioning of incisors was less obvious in our study than in prior ones, it is possible, that an alternation in toungue-lip-pressure balance or the adaption of the toungue in the agenesis region is resopnsible for this phenomen, as it was suggested before.

A further aspect, that seems to have not been investigated yet is the mandibular growth pattern calculated according to Björk [22]. On the basis of six morphological characteristics, three objective and three subjective, analyzed in lateral encephalographs the mandibular growth pattern can be described by the jaw's rotation and translation. This study confirmed that in persons with hypodontia neither a vertical nor a horizontal growth pattern is dominant. Similar analysis by Bauer et al. [18] following Hasund's method [21] revealed rather horizontal patterns in persons with missing premolars. In our group premolars were most frequently missing, however no horizontal tendency could be observed. Altough the Hasund Index [21] significantly increased, indicating a horizontal growth pattern, this was compensated by an increased inclination of the symphysis in the maijority of persons, typical for a vertical groeth pattern. There is also the fact that the facial axis, and the Jarabak index, parametres also used for growth pattern analyses, laid within the normal ranges, confirming indifferent growth patterns. It seems that hypodontia has little effect on the general mandibular growth direction.

## Conclusions

The present study reveals several significant differences in craniofacial morphology between individuals with two or more congenitally missing teeth in one jaw and norms, evaluated in persons with complete dentition. Apart from a reduction in the lower anterior facial height, we observed bimaxillary retrognathism, an increased overbite and a slightly increased overjet. Investigations in our subgroups revealed, that for some parameters it does play a role, wheter hypodontia is found in the maxilla, the mandible or in both jaws. While the reduction of the lower anterior facial height and the increased overbite were the most consistant findings, retrognathism of a jaw was primarily found, when this jaw was concerned from tooth agenesis. This indicates a connection between agenetic teeth and a saggital underdevelopment of a jaw.

Considering all results it can be concluded, that there is no predominace neither of the vertical nor the horizontal mandibular growth pattern in persons with hypodontia and, that effects of this anomaly on the craniofacial morphology are limited to a few characteristics. However, these findings need to receive special attention in orthodontic treatment of hypodontia patients and further can be urgent treatment indications themselves, such as deep bite situations.

## Competing interests

The authors declare that they have no competing interests.

## Authors' contributions

AK examined the craniofacial structures of the study and measured the orthopantomograms, lateral cephalometric radiographs. She also drafted the manuscript.

PP helped to draft the manuscript and CR made the statistical analysis. AF conceived of the study, and participated in its design and coordination and helped to draft the manuscript.

All authors read and approved the final manuscript.
